# Clinical burden of obstructive hypertrophic cardiomyopathy in France

**DOI:** 10.3389/fcvm.2024.1458410

**Published:** 2025-01-22

**Authors:** Philippe Charron, Carla Zema, François-Emery Cotté, Eléonore Herquelot, Taryn Krause, Florent Daydé, Jean-Noël Trochu

**Affiliations:** ^1^ Departments of Genetics and Cardiology, Sorbonne Université, AP-HP, Referral Center for Cardiac Hereditary Diseases, Pitié-Salpêtrière University Hospital, IHU-ICAN, INSERM UMRS_1166, Paris, France; ^2^ Health Economics and Outcomes Research, Bristol Myers Squibb, Princeton, NJ, United States; ^3^ Health Economics and Outcomes Research, Bristol Myers Squibb, Rueil-Malmaison, France; ^4^ Biostatistics, Heva, Lyon, France; ^5^ Health Economics and Outcomes Research, Bristol Myers Squibb, Uxbridge, United Kingdom; ^6^ Department of Cardiology and Vascular Diseases, Institut du Thorax, University Hospital of Nantes, University of Nantes, CNRS, INSERM, Nantes, France

**Keywords:** hypertrophic cardiomyopathy, epidemiology, NYHA, β-blockers, calcium-channel blockers, septal reduction therapy

## Abstract

**Background:**

Hypertrophic cardiomyopathy (HCM) can be genetic and occurs as obstructive and non-obstructive 21 subtypes. Little is known about the clinical burden of the obstructive subtype of HCM at a national 22 level, and how it may differ by New York Heart Association (NYHA) class. Therefore, this study 23 aimed to describe the clinical burden of patients hospitalized with obstructive HCM from a 24 nationwide study in France.

**Methods:**

This retrospective, longitudinal, observational study was performed using data from the French National Health Data System. All adult patients (≥ 18 years old) with a hospitalization related to obstructive HCM [International Classification of Diseases, Tenth Revision (ICD-10) code I42.1], or at least one hospitalization with ICD-10 code I42.2 or I42.9 and at least one code for septal reduction therapy between 2012 and 2018 were included. Patients were followed up for a minimum of 1 year. NYHA class was assigned using an algorithm based on treatment and symptom codes. Treatment patterns and clinical outcomes by NYHA class over time were examined.

**Results:**

Overall, 6,823 patients with obstructive HCM were included (54.7% male, mean [standard deviation (SD)] age 66.2 [16.7] years). At inclusion, the proportion of patients in NYHA classes I, II, III, and IV were 4%, 32%, 60%, and 4%, respectively. Over the follow-up [mean (SD) follow-up: 4.4 (2.5) years; cumulative patient follow-up: 30,021 patient-years], 73% of patients remained in the same NYHA class, 14% of patients worsened, and 13% improved. At inclusion, 22% of patients had no HCM-related treatment, 56% were receiving β-blockers, 12% calcium-channel blockers, and 11% a combination of both. The incidence of cardiovascular-related hospitalization was high (35,436 hospitalizations; 117,229 per 100,000 patient-years) and this risk increased with NYHA class (from 81,247 per 100,000 patient-years for NYHA class I/II patients to 140,790 per 100,000 patient-years for NYHA class III/IV patients, *p* < 0.0001).

**Conclusions:**

Patients with obstructive HCM are at high risk of death and cardiovascular outcomes, especially those in higher NYHA classes. Despite current therapeutics, the clinical burden of symptomatic obstructive HCM remains high, supporting the need for additional therapies.

## 1 Introduction

Hypertrophic cardiomyopathy (HCM) is a cardiac disease characterized by left ventricular hypertrophy that cannot be explained solely by loading conditions, and it can be hereditary (1–7). It is the most frequent cause of sudden death in young people and can lead to functional disability from heart failure and stroke (8). HCM occurs as obstructive and non-obstructive subtypes, which differ in symptoms, impact on quality of life, and treatment. Of those with diagnosed HCM, approximately two-thirds have obstructive HCM (2). Obstructive HCM is defined as HCM with left ventricular wall thickness of at least 15 mm in any myocardial segment, or at least 13 mm in adults with a family history of HCM, and left ventricular outflow tract obstruction (maximal gradient ≥30 mm Hg which can be resting or provoked) (2–6).

Traditionally, pharmacological therapy was used to improve functional capacity and to reduce symptoms; however, usage of such therapies is based on limited evidence (9–12) [i.e., defined by the European Society of Cardiology (ESC) as level B or above] (4). Non-vasodilating β-blockers (BB) are considered to be first-line therapy but calcium-channel blockers (CCB; e.g., verapamil, diltiazem) may be used as alternatives when BB therapy is contraindicated or does not work (4, 10). If HCM does not respond to BB therapy, combination therapy with disopyramide may be used when patients are still symptomatic; however, disopyramide is associated with known supply and access issues, and for countries where it is reimbursed, use has recently been limited and it has a poor safety profile (13, 14).

Mavacamten, a first-in-class cardiac myosin inhibitor that reduces contractility and improves myocardial energetics, is now recommended as second-line therapy by the ESC (4, 15–18) and was approved by the European Medicines Agency in June 2023 (19). As a last-line option, septal reduction therapies (SRTs) such as septal myectomy or alcohol septal ablation may be used. However, although SRTs provide definitive therapy for symptomatic left ventricular outflow tract obstruction for indicated patients by treating the obstruction (20), these procedures have potentially serious adverse events (including death) (21–28).

Previous studies have estimated the clinical burden of HCM (29–34), including one in France in 2008–2015 (35) that examined inpatient burden and showed that cardiomyopathies constitute an important cause of hospitalization, with increasing invasive therapeutic procedures and decreasing mortality; however, less is known about the overall clinical burden, especially of obstructive HCM specifically (36, 37), as well as how the disease and associated outcomes differ by New York Heart Association (NYHA) class (38). Furthermore, these studies tended to be small and subject to potential selection bias, thus, population-based studies are needed to confirm previous findings.

This study aimed to describe the clinical burden of patients hospitalized with obstructive HCM in France and to examine the outcomes by NYHA class, using a national comprehensive claims database that addresses limitations that exist in the previous evidence base. Specifically, the aims were to describe patients’ unmet needs based on patients’ medical treatment and the natural history of the disease during study follow-up.

## 2 Methods

### 2.1 Data source

This retrospective longitudinal observational study was performed using secondary pseudonymized data from the National Health Data System (SNDS), which collects individual-level data from beneficiaries of almost all health insurance schemes, covering more than 99% of the population living in France (39). The SNDS contains sociodemographic data (age, sex, and area) and information on all healthcare expenses in the community [*Données de Consommation Inter-Régimes* (DCIR)], as well as in all hospitals and healthcare facilities [*Programme de Médicalisation des Systèmes d’Information* (PMSI)] (39). Although the SNDS does not collect the reason for healthcare use, diagnoses associated with a claim, or the dosage of prescribed treatments, the DCIR reports the presence of “long-term diseases” (*Affection de Longue Durée*) and the PMSI provides the diagnosis that led to the hospital admission.

### 2.2 Inclusion criteria (clinical codes)

All adult patients (≥ 18 years old) with a hospital stay related to obstructive HCM [International Classification of Diseases, 10th Revision (ICD-10) code I42.1], or at least one hospital stay with ICD-10 code I42.2 (‘Other HCM’) or I42.9 (‘Cardiomyopathy, unspecified’) and at least one code for SRT between January 1, 2012, and December 31, 2018, were included. Patients with aortic stenosis, hypertensive heart disease, storage disease, amyloidosis, or less than 1 year of follow-up in the PMSI were excluded as these conditions could also present with left ventricular outflow obstruction and hypertrophy.

The index date was defined as the first date of obstructive HCM information in the database. Patients were followed up until the end of the study (December 31, 2019) or death, whichever occurred first.

### 2.3 Covariates

Demographic characteristics (age and sex) and Charlson Comorbidity Index (CCI) score (23) were assessed at index date. Medical history was examined over the 2 years before the index date, with major health events captured throughout the follow-up period. Health conditions were identified using specific algorithms developed by the French National Health Insurance (hypertension, coronary artery disease, heart failure, diabetes, hypercholesterolemia) or in conjunction with several expert cardiologist (myocardial infarction, cardiac dysrhythmia, atrial fibrillation, conduction disorders, pacemaker, cardiac arrest, stroke, deep vein thrombosis, pulmonary embolism, depression). These algorithms were based on specific ICD-10 codes from hospitalizations and/or LTD, specific treatments or medical acts (40). Patients’ treatments were identified 6 weeks before the index date as well as during the entirety of the follow-up period. HCM-related treatment and health outcomes were captured during the follow-up period.

### 2.4 Identification of NYHA functional class

Clinical assessment of the NYHA functional class is not available in the database, a limitation that exists in many otherwise exhaustive databases. A feasibility analysis performed in the UK CPRD database established that only 1.26% of HCM patients had a NYHA classification recorded within contributing primary care datasets. To address this limitation, an algorithm was developed utilizing treatments and symptom codes, developed in conjunction with two expert cardiologists in the UK.

The proxy algorithm retrospectively assigned a NYHA functional class to a patient both at index date (baseline) and longitudinally, over the duration of a patient's follow-up, reflecting changes over time (time-varying). The algorithm, was developed under a stepwise decision-tree method, assigning NYHA codes prioritized based on relative certainty of the data to the functional classification. Any NYHA codes in the data were prioritized in the first step; for patients without a NYHA code, assignment was subsequently based on guideline indicated treatments for heart failure, followed by treatments indicated for obstructive HCM. Patients not treated with either branch of medication were assumed to be class I so that all patients were assigned with a NYHA class as part of the algorithm. An adjustment higher was possible dependent on the presence of symptom codes which denotes a more severe symptomatic profile. The baseline NYHA class was assumed to be static over the first 3 months (post obstructive HCM diagnosis) to reflect any volatility based on initial care interventions, with NYHA allowed to fluctuate daily from 3 months onwards, based on the same decision tree utilizing data from a rolling 3-month window. Health outcomes were assigned to patients’ NYHA class at the time the event took place.

### 2.5 Statistical analyses

Continuous data were summarized by their mean and standard deviation (SD). Categorical data were summarized by frequency and percentage. The results are presented for the whole study population and by current estimated NYHA class. A Z-score based on Poisson distribution was calculated to compare the incidence of major health events during follow-up by NYHA class (NYHA classes I/II vs. NYHA classes III/IV). No sample size calculation was performed to reflect the study scope, the entirety of patients matching the inclusion criteria in France. A machine-learning technique was used to identify, group, and visualize treatment sequences (25). In brief, patients were initially sorted according to first continuous BB treatment duration. Then, a clustering technique [Time sequence Analysis through K-clustering (TAK)] was applied. Patients’ treatment sequences (vectors) were clustered using an unsupervised hierarchical Ward's clustering method (26). TAK was conducted in three main stages plus a fourth stage when each patient group detected by TAK is described. First, a matrix of pairwise distances among all patients was computed. Second, the Ward's linkage method was used to build patient clusters with the goal of minimizing the distance between patients in the cluster. The third step was to filter noise and artifacts to polish the visual output. In the output, each patient is represented by one line and the color of the line represents the treatment (or absence of treatment).

### 2.6 Ethical approvals

In accordance with the regulations in force, individual patient consent was not necessary because this is a retrospective study using only de-identified administrative data. The protection of patients’ rights and freedom was guaranteed. The study protocol obtained approval from the ethics and scientific committee for research, studies, and evaluations in the field of health (Comité éthique et scientifique pour les recherches, les études et les évaluations dans le domaine de la santé; file 1912776bis on October 8, 2020). The authorization to use the data was granted by the French data protection authority (Commission Nationale de l’Informatique et des Libertés; decision DR-2020–373 and authorization number 920414).

## 3 Results

**Figure 1:**
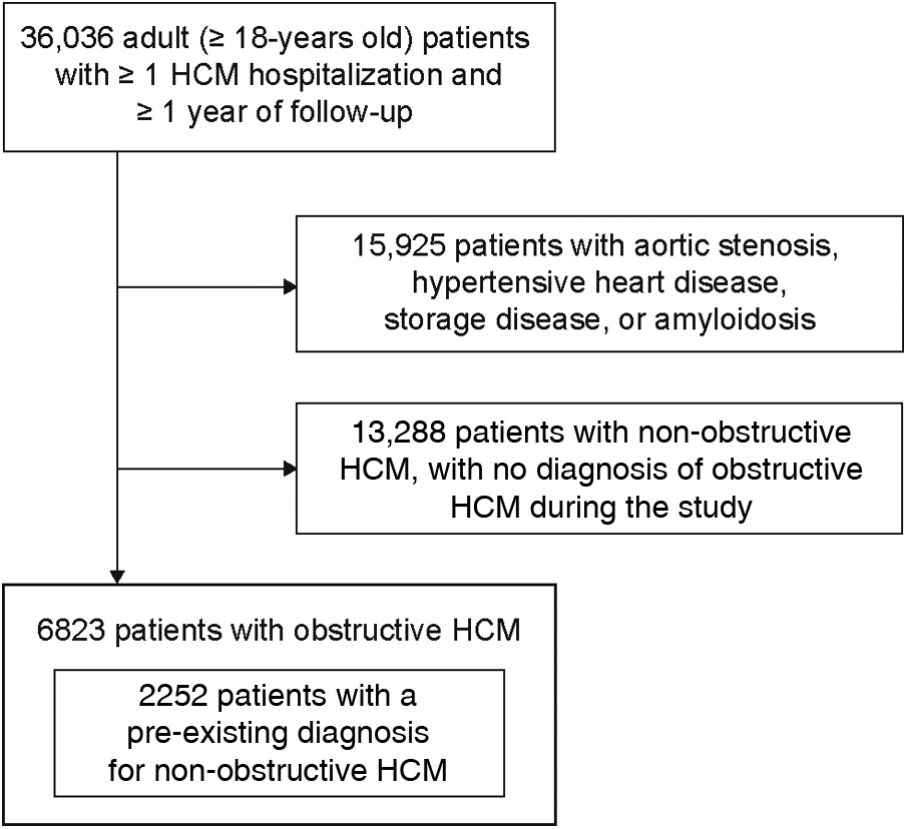
Study population selection process. HCM, hypertrophic cardiomyopathy.

Overall, 6,823 patients with obstructive HCM were included in the study, including 2,252 patients who had a pre-existing diagnosis for non-obstructive HCM (ICD-10 code I42.2 or I42.9 without a code for SRT) prior to a diagnosis for obstructive HCM (Figure 1). The mean [standard deviation (SD)] follow-up duration was 4.4 (2.5) years, corresponding to a cumulative patient follow-up of 30,021 patient-years.

### 3.1 Baseline patient characteristics

Table 1 presents the clinical characteristics of the study group at index date: 54.7% were men and the mean (SD) age was 66.2 (16.7) years. The distribution of patients by age group and sex is shown in Supplementary Figure 1; there was a larger proportion of women than men in the oldest age groups. Patients had a mean (SD) score of 3.8 (2.3) on the CCI, a standardized scoring system to capture comorbidity burden. Regarding associated cardiovascular risk factors, atrial fibrillation/flutter was present in 27.5% of patients, heart failure in 24.5%, coronary artery disease in 17.1%, and hypertension in 86.0%. Diabetes was present in 19.1% of patients and hypercholesterolemia in 9.0%. The proportion of patients with a prior SRT was 0.3%, 4.9% of patients had a pacemaker and/or implantable cardioverter-defibrillator, and 0.7% of patients had a history of cardiac arrest.

**Table 1:** Baseline patient characteristics by NYHA class.

Variable	Obstructive HCM (*N* = 6,823)	NYHA class I (*n* = 294)	NYHA class II (*n* = 2,150)	NYHA class III (*n* = 4,102)	NYHA class IV (*n* = 277)
Men, *n* (%)	3,730 (54.7)	173 (58.8)	1,301 (60.5)	2,097 (51.1)	159 (57.4)
Age, mean (SD), years	66.2 (16.7)	56.9 (21.8)	59.6 (16.8)	70.3 (14.7)	67.2 (15.8)
CCI score, mean (SD)^a^	3.8 (2.3)	2.7 (2.4)	2.8 (2.1)	4.5 (2.2)	4.6 (2.1)
Cardiovascular comorbidities, *n* (%)					
Hypertension	5,869 (86.0)	63 (21.4)	1,739 (80.9)	3,800 (92.6)	267 (96.4)
Coronary artery disease	1,169 (17.1)	21 (7.1)	322 (15.0)	754 (18.4)	72 (26.0)
Myocardial infarction	170 (2.5)	6 (2.0)	47 (2.2)	108 (2.6)	9 (3.3)
Cardiac dysrhythmia^b^	2,292 (33.6)	29 (9.9)	445 (20.7)	1,693 (41.3)	125 (45.1)
Atrial fibrillation/flutter	1,876 (27.5)	17 (5.8)	278 (12.9)	1,478 (36.0)	103 (37.2)
Heart failure^c^	1,674 (24.5)	14 (4.7)	204 (9.5)	1,281 (31.2)	175 (63.2)
Conduction disorder^d^	593 (8.7)	10 (3.4)	138 (6.4)	403 (9.8)	42 (15.2)
Pacemaker/implantable cardioverter-defibrillator	337 (4.9)	4 (1.4)	79 (3.7)	238 (5.8)	16 (5.8)
Cardiac arrest	45 (0.7)	0 (0.0)	16 (0.7)	25 (0.6)	4 (1.4)
Previous SRT	18 (0.3)	1 (0.3)	6 (0.3)	10 (0.2)	1 (0.4)
Other comorbidities, *n* (%)					
Diabetes	1,304 (19.1)	28 (9.5)	289 (13.4)	913 (22.3)	74 (26.7)
Hypercholesterolemia	614 (9.0)	11 (3.7)	160 (7.4)	411 (10.0)	32 (11.6)
Stroke	417 (6.1)	14 (4.8)	113 (5.3)	277 (6.8)	13 (4.7)
Deep vein thrombosis/pulmonary embolism	123 (1.8)	5 (1.7)	34 (1.6)	78 (1.9)	6 (2.2)
Depression	1,091 (16.0)	38 (12.9)	322 (15.0)	694 (16.9)	37 (13.4)

Baseline patient characteristics were assessed at index date (date of first obstructive HCM diagnosis in the study period) and up to 2 years prior. AIDS, acquired immune deficiency syndrome; CCI, Charlson comorbidity index; HCM, hypertrophic cardiomyopathy; HIV, human immunodeficiency virus; ICD-10, International Classification of Diseases, 10th Revision; NYHA, New York Heart Association; SD, standard deviation; SRT, septal reduction therapy.

^a^ CCI includes: myocardial infarction, heart failure, peripheral vascular disease, cerebrovascular disease, dementia, chronic lung disease, connective tissue disease, ulcerative pathology, slight liver pathology, diabetes with no complications, hemiplegia, moderate to severe renal disease, diabetes with complications, cancer, moderate or severe liver disease, metastatic disease, and HIV-AIDS.

^b^ Defined by ICD-10 I46–49, and includes conditions such as cardiac arrest, tachycardia, atrial and ventricular fibrillation, and premature depolarization.

^c^ Includes acute heart failure (PMSI ICD-10 of heart failure (I50) as principal diagnosis or ICD-10 I50 as RD or SAD with a PD of heart failure complications) and chronic heart failure (long term ICD-10 codes of heart failure (I50); hypertensive heart disease (I11), hypertensive heart and chronic kidney failure (I13) or PMSI ICD-10 of heart failure (I50) as principal diagnosis or ICD-10 I50 as RD or SAD with a PD of heart failure complications).

^d^ Defined by ICD-10 I44–45, and includes conditions such as atrioventricular block, fascicular block, and bundle-branch block.

At index date, the proportions of patients in NYHA classes I, II, III, and IV were 4% (294 patients), 32% (2,150 patients), 60% (4,102 patients), and 4% (277 patients), respectively. Aside from NYHA class IV, higher age was observed in higher NYHA classes. As NYHA class increased, the mean CCI score increased from 2.7 (NYHA class I) to 4.6 (NYHA class IV), and so did the proportion of patients with cardiovascular comorbidities. In NYHA class IV (vs. NYHA class I), atrial fibrillation/flutter was present in 37.2% of patients (vs. 5.8%), heart failure in 63.2% (vs. 4.7%), and coronary artery disease in 26.0% (vs. 7.1%), and 5.8% of patients had a pacemaker and/or implantable cardioverter-defibrillator (vs. 1.4%). Regarding associated cardiovascular risk factors in NYHA class IV (vs. NYHA class I), diabetes was present in 26.7% of patients (vs. 9.5%), hypercholesterolemia in 11.6% (vs. 3.7%), and hypertension in 96.4% (vs. 21.4%).

### 3.2 Pharmacological treatment dispositions and patterns in patients with obstructive HCM

**Figure 2:**
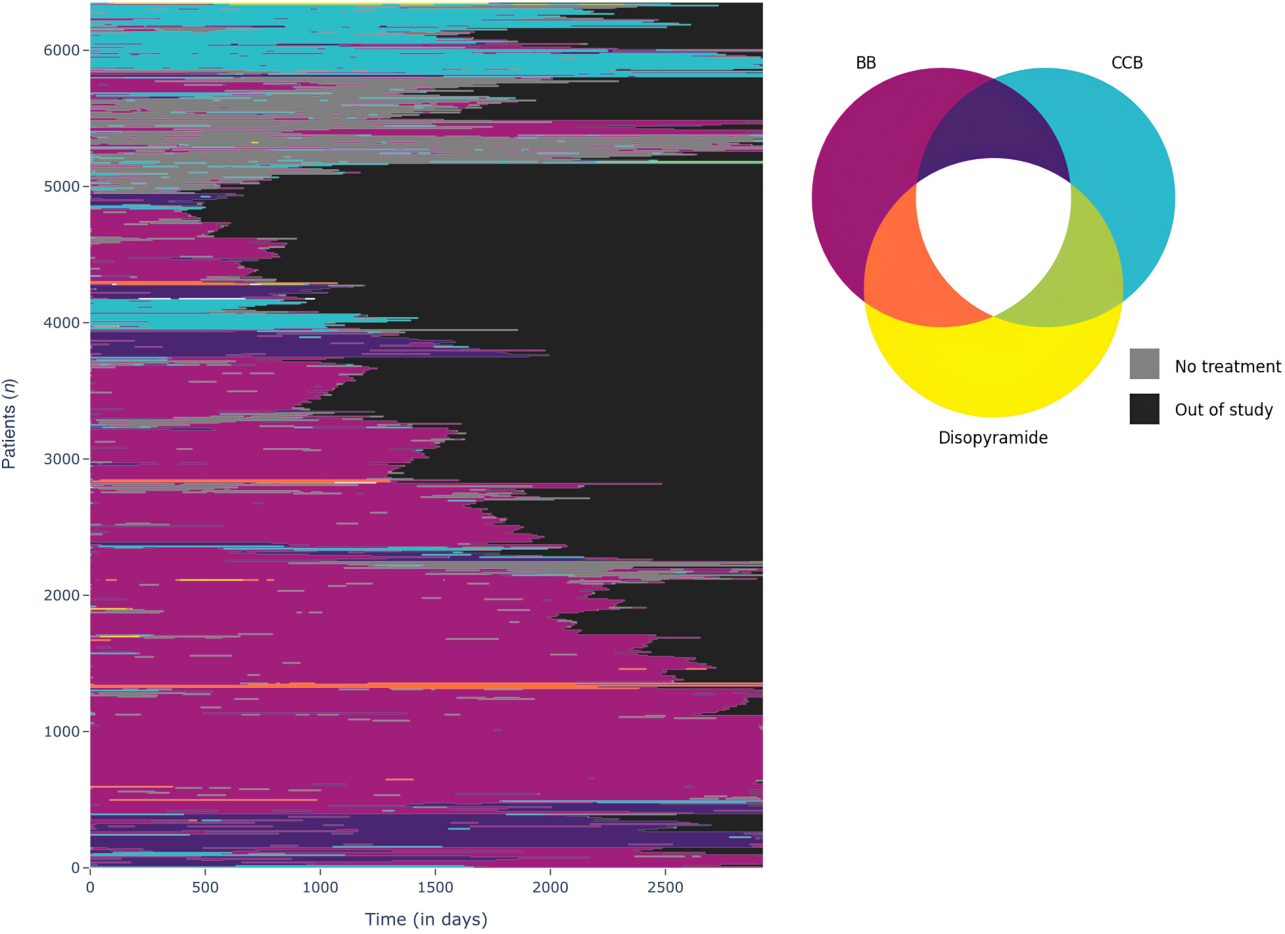
Patient pharmacological treatment sequences from index date to end of follow-up. Figure computed via a machine-learning technique to identify group and to visualize treatment sequences (Time sequence Analysis through K-clustering). In the output, each patient is represented by one line and the color of the line represents the treatment (or absence of treatment). BB, β-blockers; CCB, calcium-channel blockers.

Figure 2 visualizes pharmacological treatment sequences within the patient population from index date to end of follow-up. At index date, 21% of patients had no HCM-related treatments. HCM-related treatments were mainly BB (56% of patients), CCB (12% of patients), or a combination of both BB and CCB (11% of patients). Disopyramide was used either as a standalone therapy or in combination with BB or CCB in 86 patients (1%).

**Figure 3:**
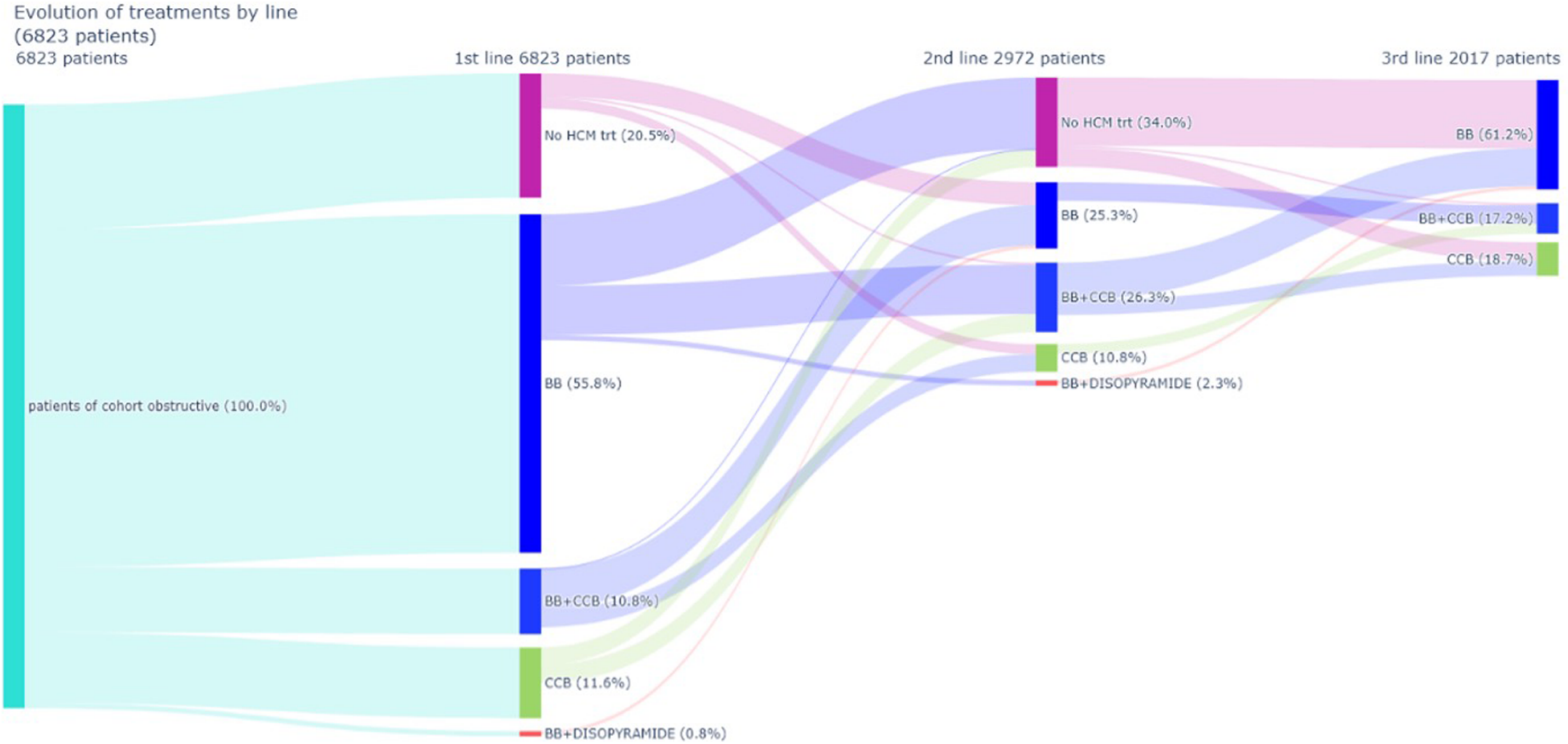
Drugs switches from index date (line 1) to line 2 and from line 2 to line 3. BB, β-blockers; CCB, calcium-channel blockers; HCM, hypertrophic cardiomyopathy.

**Figure 4:**
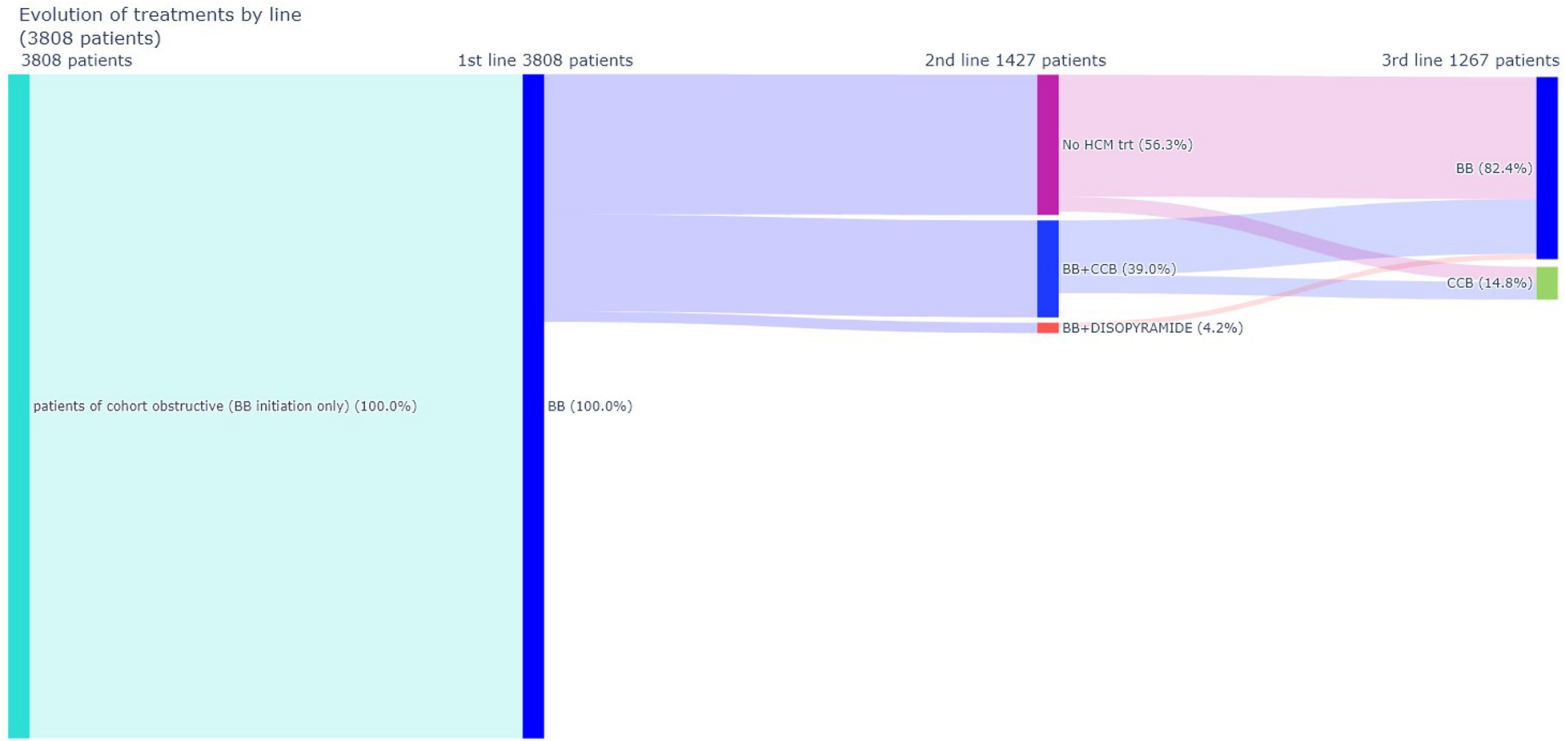
Drugs switches from index date (line 1) to line 2 and from line 2 to line 3 (BB monotherapy at index date subpopulation). BB, β-blockers; CCB, calcium-channel blockers; HCM, hypertrophic cardiomyopathy.

The analysis of treatment trajectories indicates that in most cases patients remained treated with their initial therapy, that is with monotherapies (BB or CCB). By contrast, approximately 1,000 patients have very discontinuous treatment sequences, alternating periods with specific treatment and periods without. Figures 3, 4 details drugs’ switches from index date by line for the study population and a subpopulation initiating a monotherapy with a BB at index date respectively. Among the 3,808 patients treated with a BB as monotherapy, 37.4% (*N* = 1,427) of patients switched to a 2nd line. Of these, 56.3% (*N* = 804) switched to no HCM specific treatments and in 39% (*N* = 556) of cases CCB was added to BB. Among patients treated at baseline by a biotherapy (CCB + BB), only 72% of patients did not switch to a second line. These patients switched to monotherapies (i.e., BB in 68.4% of cases and CCB in 29.8% of cases). For patients initiating a CCB in line 1, 51.2% (*N* = 407) switched to a 2nd line, mainly to no HCM-related treatments (47.7%) or for a biotherapy including BB (49.6%). For patients initiating disopyramide, 12/13 patients switched to a 2nd line, mainly to CCB.

**Figure 5:**
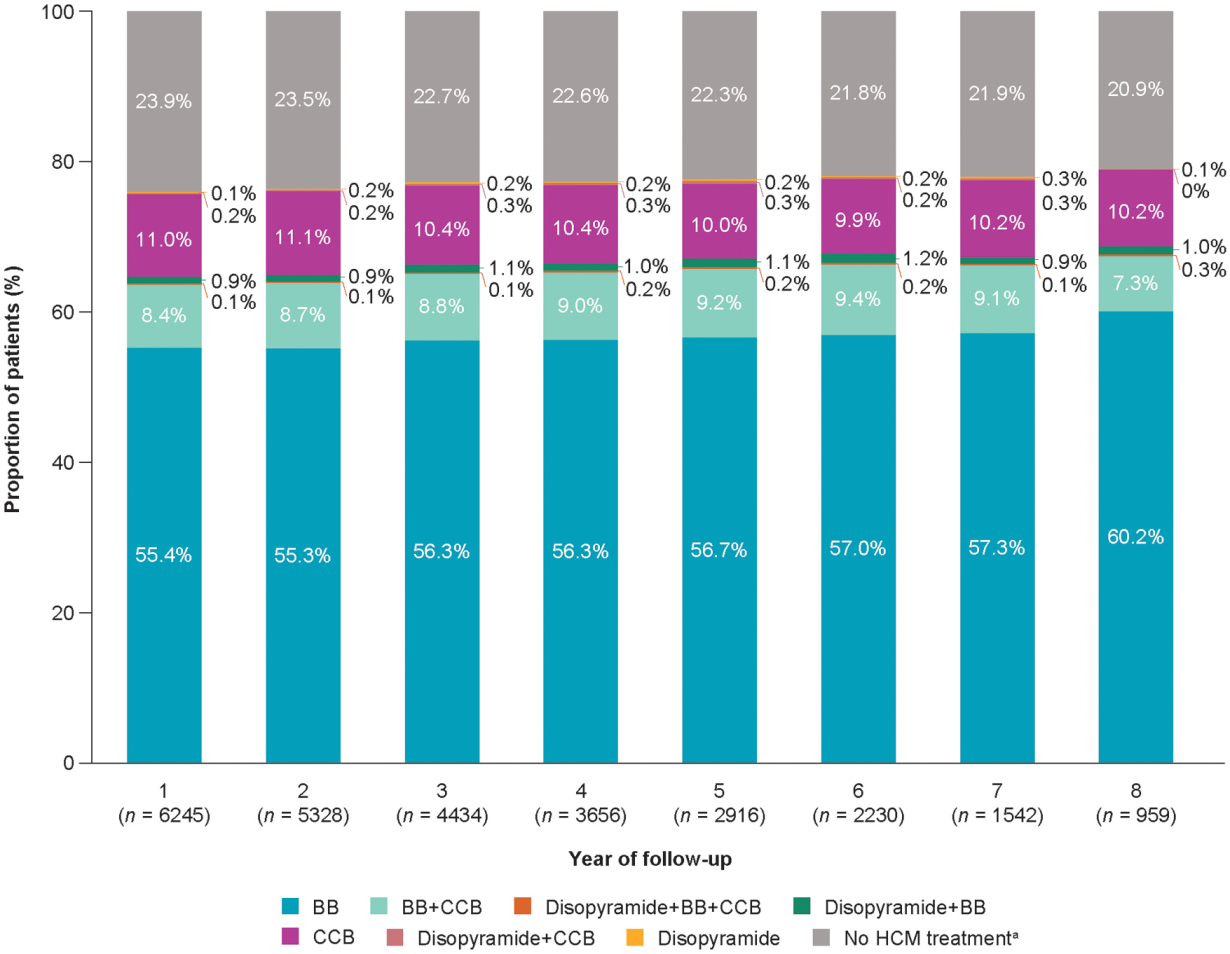
Patient pharmacological treatment distributions from index date to end of follow-up. Patient pharmacological treatment trajectories from index date to end of follow-up. Percentages are based on the included patients in each analysis year. ^a^No BB, CCB, or disopyramide treatments. BB, β-blockers; CCB, calcium-channel blockers; HCM, hypertrophic cardiomyopathy.

Patients generally remained on the same therapy during study follow-up (Figures 2, 3). As detailed in the Figure 5, BB were the most frequently delivered therapy (range, 55.4–60.2%) irrespective of the year of follow-up. Disopyramide was used either as a standalone therapy or in combination with BB or CCB in 227 patients (3.3%).

### 3.3 Health outcomes

Overall and during follow-up [mean [SD] follow-up duration, 4.4 [2.5] years], 73% of patients remained in the same NYHA class, 14% worsened (increase of NYHA class), and only 13% improved (decrease of NYHA class) (Table 2, Supplementary Figure 2).

**Table 2:** NYHA class evolution during the follow-up, stratified by baseline NYHA.

Year	Baseline NYHA class I (*n* = 294)					Baseline NYHA class II (*n* = 2,150)				
	Patients, *n*^a^	NYHA class at follow-up, *n*				Patients, *n*^a^	NYHA class at follow-up, *n*			
		I	II	III	IV		I	II	III	IV
1^b^	283	283	0	0	0	2,002	0	2,002	0	0
2	236	184	37	15	0	1,767	15	1,524	226	2
3	193	130	38	25	0	1,531	2	1,210	314	5
4	155	79	50	26	0	1,323	3	973	344	3
5	124	56	46	22	0	1,101	1	754	339	7
6	101	45	41	15	0	859	2	567	284	6
7	75	29	32	14	0	598	0	390	201	7
Year	Baseline NYHA class III **(*n* = 4,102)**					Baseline NYHA class IV **(*n* = 277)**				
	Patients, ***n*^a^**	NYHA class at follow-up, ***n***				Patients, ***n*^a^**	NYHA class at follow-up, ***n***			
		I	II	III	IV		I	II	III	IV
1^b^	3,697	0	0	3,697	0	262	0	0	0	262
2	3,099	7	437	2,604	51	214	0	11	123	80
3	2,538	3	402	2,088	45	161	0	7	104	50
4	2,036	1	364	1,616	55	129	0	11	86	32
5	1,578	2	326	1,215	35	105	0	8	72	25
6	1,187	0	267	893	27	78	0	9	53	16
7	808	3	178	611	16	57	0	6	39	12

HCM, hypertrophic cardiomyopathy; NYHA, New York Heart Association.

^a^ Represents the number of patients still in the study at the given time point.

^b^ Transitions in the first year were not captured.

**Figure 6:**
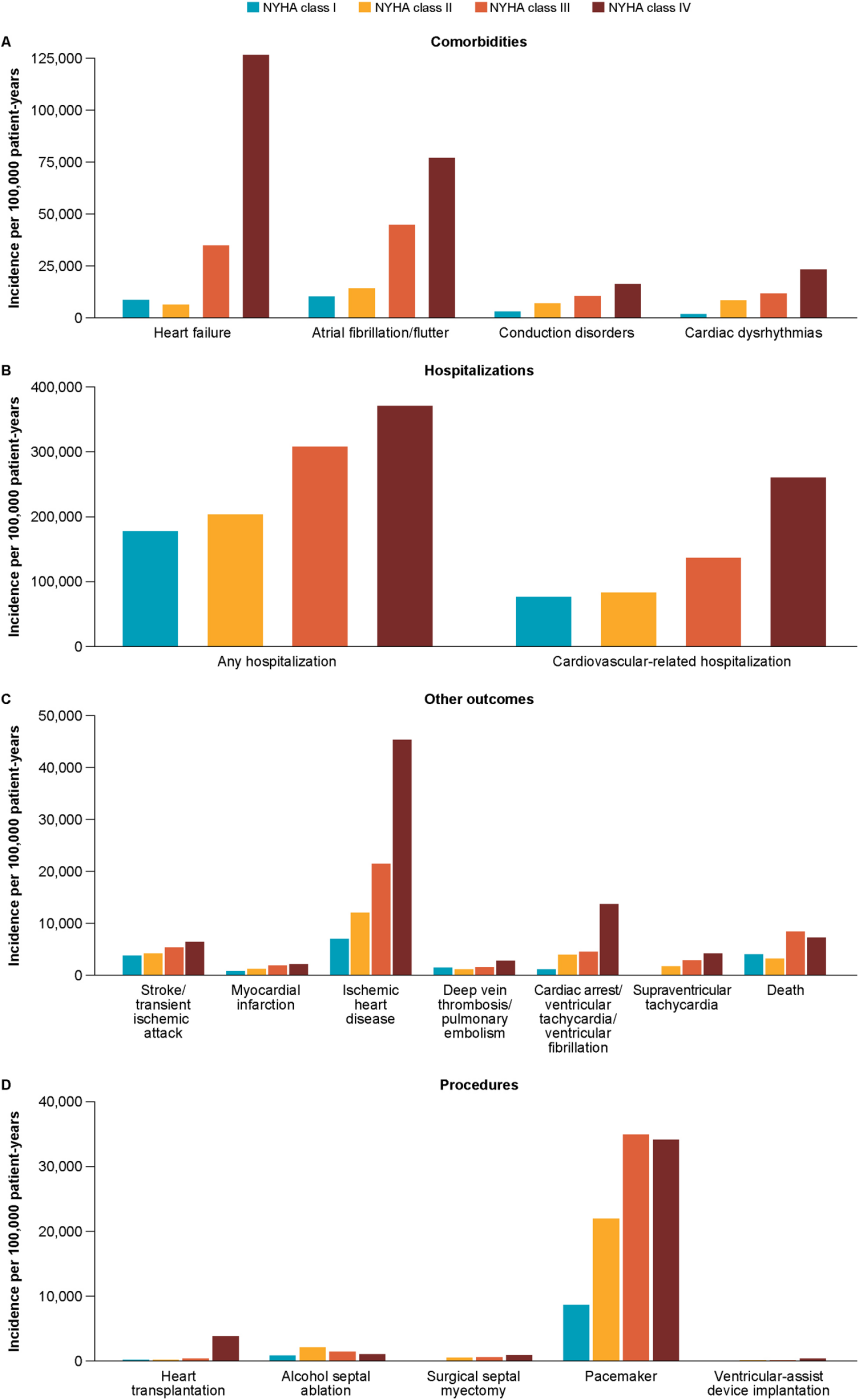
Incidence of health outcomes (**A**: comorbidities; **B**: hospitalizations; **C**: other outcomes; **D**: procedures) by NYHA class. NYHA, New York Heart Association.

Among study patients, 1,886 patients died during follow-up (6,239 deaths per 100,000 patient-years). The number of heart transplantations was 102 (337 transplantations per 100,000 patient-years), and the incidence of cardiovascular-related hospitalization was high (35,436 hospitalizations; 117,229 per 100,000 patient-years). The incidence of SRTs was 2,107 per 100,000 patient-years, with alcohol septal ablation accounting for 77% (*n* = 492) of all SRT. Figure 6 and Supplementary Table 1 depicts the incidence of health outcomes for the overall cohort.

As detailed in Table 3 and Supplementary Table 2, the incidence of health outcomes increased with NYHA class during follow-up. The incidences of hospitalizations, cardiovascular-related hospitalizations, major cardiovascular adverse events, heart failure, atrial fibrillation/flutter, conduction disorders, pacemaker/implantable cardioverter-defibrillator, and heart transplantation were always significantly higher in NYHA classes III/IV than in NYHA classes I/II (*p* < 0.0001 for all comparisons). In NYHA classes III/IV (vs. NYHA classes I/II), the incidences per 100,000 patient-years observed for the following major outcomes were: 140,790 cardiovascular-related hospitalizations (vs. 81,247), 8,255.4 deaths (vs. 3,160.3), 37,447 heart failure cases (vs. 6,110.1), 45,869 atrial fibrillation/flutter cases (vs. 13,639), and 514.59 heart transplantations (vs. 66.88).

**Table 3:** Comparisons of incidence of health outcomes by aggregated NYHA class for obstructive HCM.

	NYHA classes I/II		NYHA classes III/IV		*p* value
	Incidence^b^	95% CI	Incidence^b^	95% CI	
Hospitalizations	200,191	197,671–202,743	309,806	307,264–312,369	< 0.0001
Cardiovascular-related hospitalizations	81,247	79,647.5–82,878.4	140,790	139,080–142,521	< 0.0001
Death	3,160.3	2,857.20–3,495.45	8,255.4	7,849.05–8,682.73	< 0.0001
MACE^a^	8,335.4	7,833.71–8,869.17	15,372	14,813.9–15,951.3	< 0.0001
Heart failure	6,110.1	5,659.64–6,596.40	37,447	36,429.6–38,492.0	< 0.0001
Atrial fibrillation/flutter	13,639	12,950.2–14,364.6	45,869	44,709.8–47,057.8	< 0.0001
Conduction disorders	6,362.3	5,925.96–6,830.79	10,577	10,115.3–11,058.8	< 0.0001
Pacemaker/implantable cardioverter-defibrillator	20,935	20,130.5–21,770.8	34,866	34,020.5–35,733.2	< 0.0001
Heart transplantation	66.88	33.45–133.74	514.59	420.41–629.88	< 0.0001
SRT	2,407.8	2,145.19–2,702.59	1,910.6	1,720.27–2,121.90	0.0037

CI, confidence interval; HCM, hypertrophic cardiomyopathy; MACE, major adverse cardiovascular events; NYHA, New York Heart Association; SRT, septal reduction therapy.

^a^ Composite of death or myocardial infarction, transient ischemic attack, or stroke of any type.

^b^ Incidence for 100,000 patient-years.

## 4 Discussion

This is the first nationwide study in France to demonstrate that patients with obstructive HCM have a high clinical burden (i.e., numerous comorbidities and high incidence of major cardiac-related outcomes). In this national, observational, retrospective study in patients hospitalized with obstructive HCM who were followed up for at least 1 year and 4.4 years on average, we observed that most patients were in NYHA class III/IV (2/3 of patients) and, irrespective of the year of follow-up BB were the most frequently delivered therapy. Thus, treatment patterns aligned with guideline-directed care in that most patients received BB, some received CCB (sometimes combined with BB), and few received disopyramide (most of the time combined with BB) (4, 11). It is interesting to note that some patients were not treated at all during the study or stopped treatment. This could be because patients did not tolerate the treatment, or symptoms were not improved, and no other treatment options were available. This observation supports the high burden of unmet therapeutic needs in obstructive HCM. Moreover, 21% of patients were not treated with specific treatment at index date. This high proportion may be explained by misdiagnosis, late diagnosis, diagnosis with a low disease burden, or patient-specific factors such as non-adherence, refusal of treatment or contraindications.

Interestingly, a major finding observed is that most patients remained in the same NYHA class during follow-up and only 13% of patients improved NYHA class, with some patients worsening despite the various therapies that could be delivered to the patients. Very few data are published in the literature about the evolution of symptoms and NYHA classes (41). A retrospective cohort study conducted in the USA using the Optum Market Clarity database linked to claims and electronic health records included 4,631 patients with obstructive HCM. This study depicted no improvement in patients in NYHA class IV during 1 year of follow-up. By contrast, an improvement was observed for 10.1% of patients in NYHA class II and 15.6% of those in NYHA class III (41).

With close to 1,900 deaths and 35,500 cardiovascular-related hospitalizations in the population, the study findings stress the high clinical burden of obstructive HCM and the substantial cardiovascular consequences of the disease. In 2015, a French study, also using the PMSI, estimated that the prevalence of HCM was 10.1 hospitalized patients (major cardiovascular events and procedures) per 100,000 patient-years (35). Our study underscores the high burdens of hospitalization and cardiovascular hospitalization, which represent important aims for new medical therapies as well as mortality reduction. This further highlights unmet therapeutic needs as already depicted in the literature (42). Several studies examined the clinical burden of obstructive HCM in Europe and in the USA. A population-based study conducted in the USA found that patients with obstructive HCM were approximately four times as likely to have any inpatient admission and cost the healthcare system nearly US$20,000 more per year than age- and sex-matched controls (43). In England, based on electronic health records from clinical practice between 2009 and 2020, the mortality was lower (1,890 deaths per 100,000 patient-years vs. 6,239 deaths per 100,000 patient-years) than that observed in the results of this study (44). In this study, however, the baseline NYHA class distribution revealed that a higher proportion of patients were in NYHA class I and fewer were in class III than in our study (26%, 34%, 38%, and 3% of patients in NYHA classes I, II, III, and IV, respectively). In Germany, using a nationally representative administrative claims data pool from several German Statutory Health Insurances in 2012–2018, the distribution of NYHA classes (43%, 24%, 29%, and 3% in NYHA classes I, II, III, and IV, respectively) showed that patients had fewer cardiovascular symptoms at baseline than those in our study (45). These results were similar to those from a multicenter international study conducted in Europe (Greece, Italy, Spain and UK) demonstrating an excess of mortality in HCM patients compared to the general population with a distinct 54.6%, 34.4% and 11% in NYHA classes I, II, and III/IV, respectively) (46). The lower proportion of asymptomatic participants in our study (NYHA class I) is probably due to the capture of diagnosis at hospital only, when the patients are managed for more severe symptoms. This constitutes a limitation of this administrative dataset. Thus, the absence of clinical data could have led to an underestimation of patients in NYHA class I and consequently a bias towards inclusion to select patients with more severe diseases. Stratifying by NYHA class allowed the demonstration of the impact of health outcomes across the class distribution.

The patients with most severe disease were found to have more comorbidities at baseline and numerous cardiovascular risk factors (hypertension, heart failure, diabetes, dyslipidemia) which could directly impact the incidence of cardiovascular complications.

A second major finding is that the burden of cardiovascular complications was high in obstructive HCM and that cardiovascular outcomes significantly increased with severity of NYHA class. The patients with most severe disease were found to have more comorbidities at baseline and numerous cardiovascular risk factors (hypertension, heart failure, diabetes, dyslipidemia) which could directly impact the incidence of cardiovascular complications. Few data in the literature focused on obstructive HCM or explored major health outcomes according to NYHA class. Results from Osman et al. were consistent with our findings; incidences of cardiovascular outcomes were generally greater in higher NYHA classes than in lower classes (28). In the USA, Wang et al. demonstrated that patients with obstructive HCM with higher NYHA classes had increased risks of a large variety of cardiovascular outcomes: all-cause mortality, all-cause hospitalization, cardiovascular-related hospitalization, primary ischemic stroke/transient ischemic attack, myocardial infarction, deep vein thrombosis/pulmonary embolism, and major adverse cardiovascular events (41).

### 4.1 Strengths and limitations

This study has several strengths. It is based on real-world data and offers quasi-national coverage. Single-center studies offer small sample sizes, thereby limiting the ability to understand the full clinical burden of obstructive HCM. Here, the large study population presented a representative burden of hospitalized obstructive HCM and allowed for the examination of outcomes by subgroups (NYHA classification).

The study also has some limitations, which should be kept in mind when interpreting the results. First, it has all the limitations of similar studies based on administrative data (e.g., absence of diagnostic test results). The quality of claims data used for this study depends on the individual completeness and quality of coding for billing purposes and on the existing classification systems. Secondly, because only patients with a hospitalization with HCM could be identified, patients with HCM who were never admitted to hospital during the study inclusion period were not included. Therefore, the results are not representative of all patients with obstructive HCM living in France. Particularly, patients with less severe symptoms (NYHA classes I and II) are underestimated in the study, while almost all patients in NYHA classes III and IV were probably included. Thirdly, NYHA class and symptoms are not directly coded in the database and patients were assigned to NYHA classes based on an algorithm developed with clinical expert input. Despite the algorithm being developed in conjunction with several expert cardiologists, there will likely be some NYHA misclassification, which has the potential to impact the assessment of outcomes. Moreover, health care databases such as these are increasingly being used to conduct observational studies, notably pharmaco-epidemiological studies, because of their many strengths including the accurate reflection of nation-level clinical practice data (e.g., inclusion of drugs, medical acts, hospitalizations) with minimal loss of follow up. In France, there are national standards of coding PMSI data, e.g., the choice of the principal diagnosis. Of course, coding practices may vary between hospitals or regions because of different interpretation of coding rules and this could not be ruled out. However, it is worth noting that quality of coding is regularly audited in public and private hospitals as part of reimbursement administration.

Finally, only patients with at least 1 year of follow-up in the PMSI (and thus still alive at 1 year and hospitalized at least 1 year apart from the index date) were included in this study. This study is possibly biased towards patients with an improved health status.

## 5 Conclusion

This nationwide real-world study in France demonstrated that hospitalized patients with obstructive HCM have a high clinical burden. Patients with obstructive HCM in higher NYHA classes were at greater risk of death, cardiovascular outcomes (including atrial fibrillation/flutter and heart failure), and hospitalization than those in lower NYHA classes. Our results suggest that the clinical burden of symptomatic obstructive HCM remains high in the context of current therapeutics, thus supporting the need for additional therapies.

## Data Availability

Publicly available datasets were analyzed in this study. This data can be found here: https://www.health-data-hub.fr.
