# Supplementary material for: Clinical burden of obstructive hypertrophic cardiomyopathy in France
Source: Front Cardiovasc Med. 2025 Jan 22;11:1458410. doi: 10.3389/fcvm.2024.1458410 (PMC11794283; doi:10.3389/fcvm.2024.1458410)

Supplementary material

SUPPLEMENTARY TABLE 1 Incidence of health outcomes overall cohort.

|  | **Obstructive HCM** | | | |
| --- | --- | --- | --- | --- |
| **Event category** | **Events, *n*** | **Patient-years^a^** | **Incidence^b^** | **95% CI** |
| Heart transplantation | 102 | 30,227.962 | 337.44 | 277.91–409.71 |
| Heart failure | 5722 | 24,251.231 | 23,595 | 22,991.2–24,214.0 |
| SRT | 637 | 30,227.962 | 2107.3 | 1949.87–2277.49 |
| Alcohol septal ablation | 492 | 30,227.962 | 1627.6 | 1489.98–1778.00 |
| Surgical septal myectomy | 146 | 30,227.962 | 483.00 | 410.67–568.05 |
| Dilated cardiomyopathy | 1377 | 28,680.608 | 4801.2 | 4554.15–5061.56 |
| Stroke/transient ischemic attack | 1463 | 30,227.962 | 4839.9 | 4598.13–5094.36 |
| Atrial fibrillation/flutter | 7295 | 23,271.053 | 31,348 | 30,636.8–32,075.6 |
| Pacemaker | 8873 | 30,227.962 | 29,354 | 28,749.2–29,970.8 |
| Myocardial infarction | 456 | 30,227.962 | 1508.5 | 1376.24–1653.55 |
| Ischemic heart disease | 5473 | 30,227.962 | 18,106 | 17632.4–18591.8 |
| Deep vein thrombosis/pulmonary embolism | 408 | 30,227.962 | 1349.7 | 1224.93–1487.28 |
| Cardiac arrest/ventricular tachycardia/ventricular fibrillation | 1316 | 30,227.962 | 4353.6 | 4124.61–4595.27 |
| Supraventricular tachycardia | 694 | 30,227.962 | 2295.9 | 2131.27–2473.21 |
| Conduction disorders | 2693 | 30,227.962 | 8909.0 | 8578.77–9251.88 |
| Cardiac dysrhythmias | 3095 | 30,227.962 | 10,239 | 9884.43–10,606.0 |
| Ventricular assist device implantation | 10 | 30,227.962 | 33.08 | 17.80–61.48 |
| Any hospitalization | 80,537 | 30,227.962 | 266,432 | 264,598–268,279 |
| Cardiovascular-related hospitalization | 35,436 | 30,227.962 | 117,229 | 116,015–118,456 |
| Death | 1886 | 30,227.962 | 6239.3 | 5963.93–6527.29 |
| Composite of death or myocardial infarction, transient ischemic attack, stroke of any type | 3805 | 30,227.962 | 12,588 | 12,194.0–12,994.1 |
| Composite of death or heart transplantation | 1988 | 30,227.962 | 6576.7 | 6293.85–6872.24 |

^a^Patient-years refers to the number of years for which patients contribute data. ^b^Incidence per 100,000 patient-years.
CI, confidence interval; HCM, hypertrophic cardiomyopathy; SRT, septal reduction therapy.

SUPPLEMENTARY TABLE 2 Incidence of health outcomes by aggregated NYHA class for obstructive HCM.

|  | **NYHA classes I/II** | | | | **NYHA classes III/IV** | | | |
| --- | --- | --- | --- | --- | --- | --- | --- | --- |
| **Event category** | **Events, *n*** | **Patient-years^a^** | **Incidence^b^** | **95% CI** | **Events, *n*** | **Patient-years^a^** | **Incidence^b^** | **95% CI** |
| Heart transplantation | 8 | 11,961.07 | 66.88 | 33.45–133.74 | 94 | 18,266.891 | 514.59 | 420.41–629.88 |
| Heart failure | 655 | 10,719.97 | 6110.1 | 5659.64–6596.40 | 5067 | 13,531.261 | 37447 | 36,429.6–38,492.0 |
| SRT | 288 | 11,961.07 | 2407.8 | 2145.19–2702.59 | 349 | 18,266.891 | 1910.6 | 1720.27–2121.90 |
| Alcohol septal ablation | 237 | 11,961.07 | 1981.4 | 1744.56–2250.45 | 255 | 18,266.891 | 1396.0 | 1234.73–1578.26 |
| Surgical septal myectomy | 51 | 11,961.07 | 426.38 | 324.05–561.04 | 95 | 18,266.891 | 520.07 | 425.33–635.90 |
| Dilated cardiomyopathy | 242 | 11,587.598 | 2088.4 | 1841.22–2368.86 | 1135 | 17,093.01 | 6640.1 | 6264.86–7037.90 |
| Stroke/transient ischemic attack | 486 | 11,961.07 | 4063.2 | 3717.53–4440.97 | 977 | 18,266.891 | 5348.5 | 5023.40–5694.59 |
| Atrial fibrillation/flutter | 1430 | 10,484.572 | 13,639 | 12,950.2–14,364.6 | 5865 | 12,786.48 | 45,869 | 44,709.8–47,057.8 |
| Pacemaker | 2504 | 11,961.07 | 20,935 | 20,130.5–21,770.8 | 6369 | 18,266.891 | 34,866 | 34,020.5–35,733.2 |
| Myocardial infarction | 133 | 11,961.07 | 1111.9 | 938.15–1317.92 | 323 | 18,266.891 | 1768.2 | 1585.53–1971.97 |
| Ischemic heart disease | 1387 | 11,961.07 | 11,596 | 11,001.5–12,222.6 | 4086 | 18,266.891 | 22,368 | 21,692.9–23,064.8 |
| Deep vein thrombosis/pulmonary embolism | 129 | 11,961.07 | 1078.5 | 907.56–1281.63 | 279 | 18,266.891 | 1527.4 | 1358.25–1717.51 |
| Cardiac arrest/ventricular tachycardia/ventricular fibrillation | 433 | 11,961.07 | 3620.1 | 3294.67–3977.63 | 883 | 18,266.891 | 4833.9 | 4525.34–5163.47 |
| Supraventricular tachycardia | 176 | 11,961.07 | 1471.4 | 1269.35–1705.71 | 518 | 18,266.891 | 2835.7 | 2601.75–3090.76 |
| Conduction disorders | 761 | 11,961.07 | 6362.3 | 5925.96–6830.79 | 1932 | 18,266.891 | 10,577 | 10,115.3–11,058.8 |
| Cardiac dysrhythmias | 906 | 11,961.07 | 7574.6 | 7097.07–8084.21 | 2189 | 18,266.891 | 11,983 | 11,491.8–12,496.1 |
| Ventricular assist device implantation | 1 | 11,961.07 | 8.36 | 1.18–59.35 | 9 | 18,266.891 | 49.27 | 25.64–94.69 |
| Any hospitalization | 23,945 | 11,961.07 | 20,0191 | 19,7671–20,2743 | 56,592 | 18,266.891 | 309,806 | 307,264–312,369 |
| Cardiovascular-related hospitalization | 9718 | 11,961.07 | 81,247 | 79,647.5–82,878.4 | 25,718 | 18,266.891 | 140,790 | 139,080–142,521 |
| Death | 378 | 11,961.07 | 3160.3 | 2857.20–3495.45 | 1508 | 18,266.891 | 8255.4 | 7849.05–8682.73 |
| Composite of death or myocardial infarction, transient ischemic attack, stroke of any type | 997 | 11,961.07 | 8335.4 | 7833.71–8869.17 | 2808 | 18,266.891 | 15,372 | 14,813.9–15,951.3 |
| Composite of death or heart transplantation | 386 | 11,961.07 | 3227.1 | 2920.74–3565.68 | 1602 | 18,266.891 | 8770.0 | 8350.86–9210.11 |

^a^Patient-years refers to the number of years for which patients contribute data. ^b^Incidence per 100,000 patient-years.
CI, confidence interval; HCM, hypertrophic cardiomyopathy; NYHA, New York Heart Association; SRT, septal reduction therapy.

# SUPPLEMENTARY FIGURE 1

Population pyramid of patients with obstructive HCM at index date. HCM, hypertrophic cardiomyopathy.


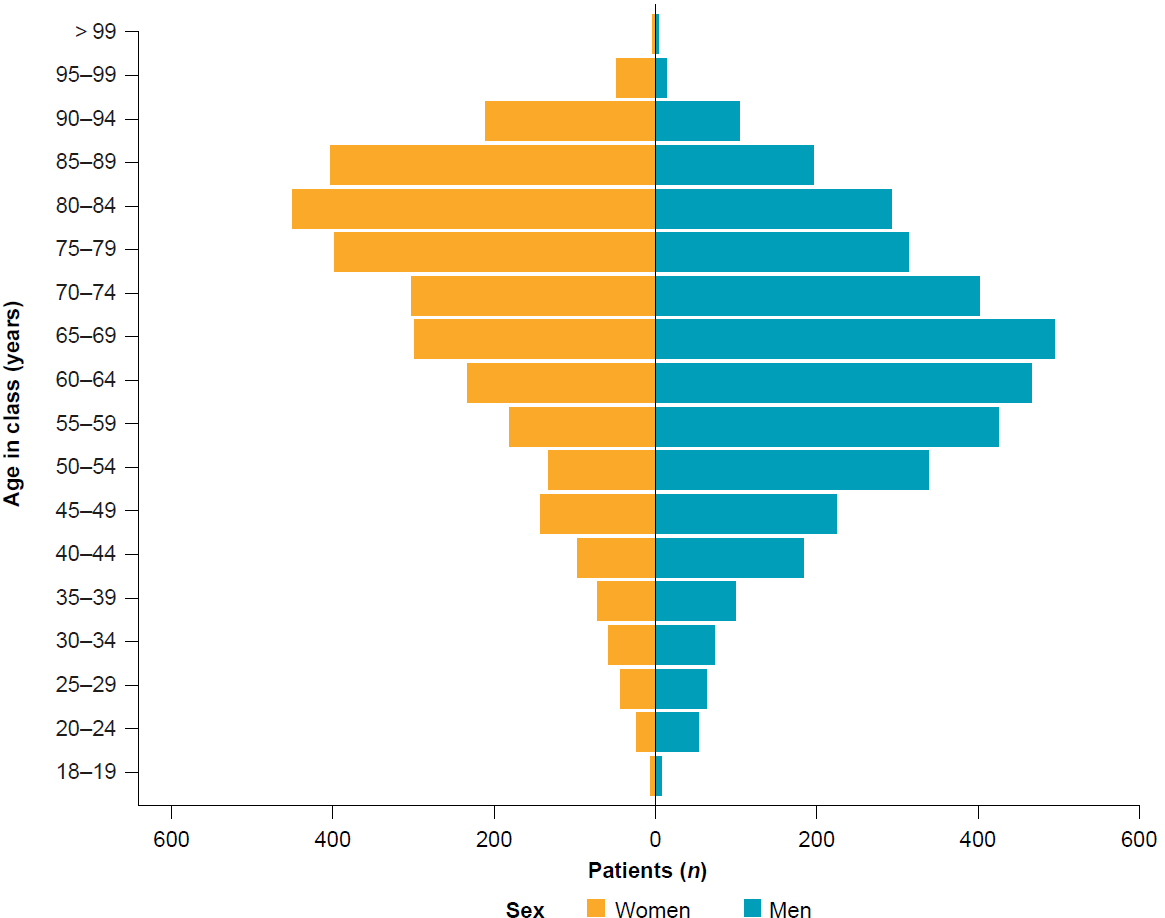


# SUPPLEMENTARY FIGURE 2

Proportion of patients in each NYHA class, and changes in NYHA class over time, by follow-up year. NYHA, New York Heart Association.


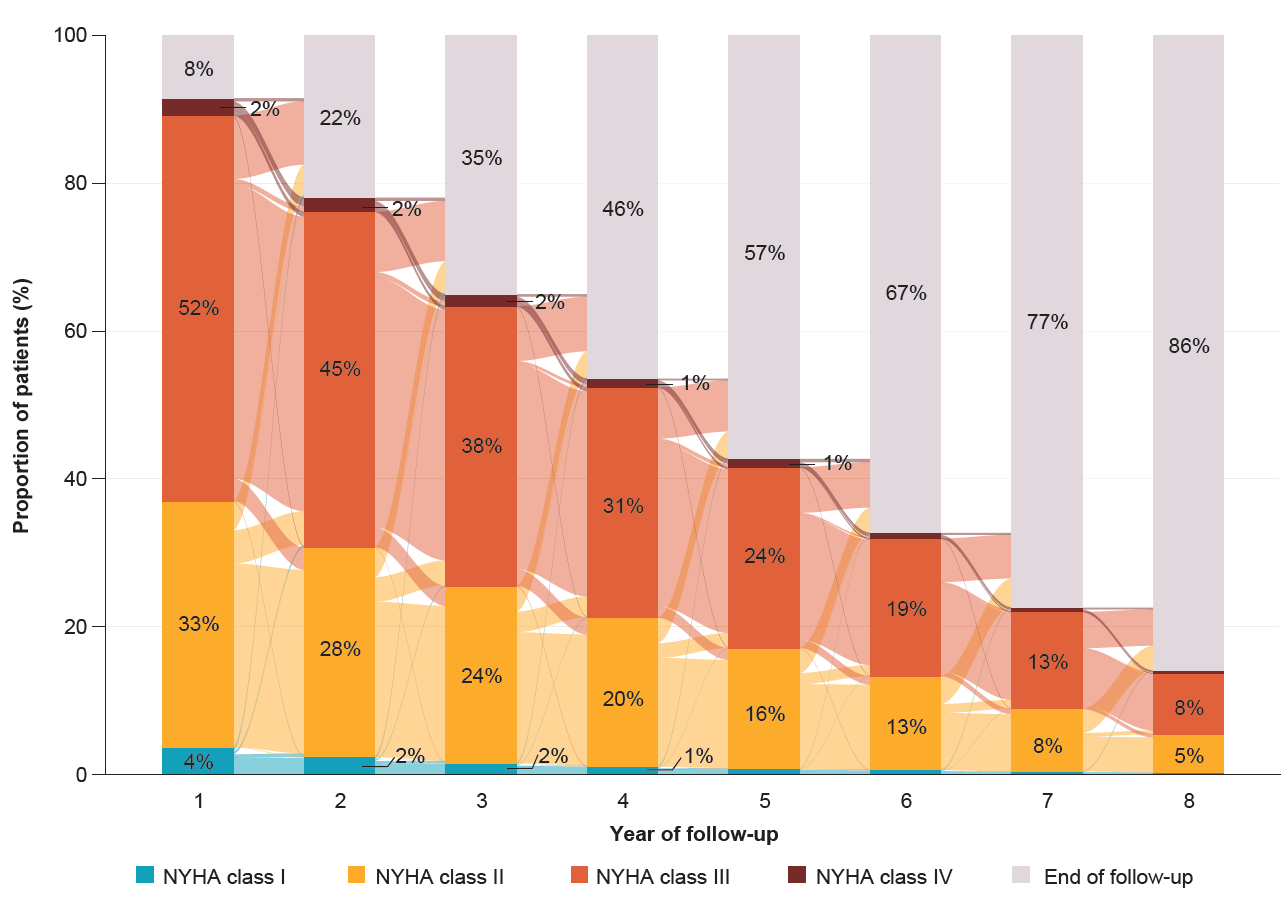

Supplement: Supplementary file 1 [file Supplementaryfile1.docx]
